# Chronic Illness Perceptions and Cardiovascular Disease Risk Behaviors in Black and Latinx Sexual Minority Men with HIV: A Cross-Sectional Analysis

**DOI:** 10.3390/nursrep14030143

**Published:** 2024-08-08

**Authors:** S. Raquel Ramos, Baram Kang, Sangchoon Jeon, Marilyn Fraser, Trace Kershaw, Mohamed Boutjdir

**Affiliations:** 1 School of Nursing, Yale University, Orange, CT 06477, USA; baram.kang@yale.edu (B.K.); sangchoon.jeon@yale.edu (S.J.); 2 School of Public Health, Social and Behavioral Sciences, Yale University, New Haven, CT 06520, USA; trace.kershaw@yale.edu; 3 Center for Interdisciplinary Research on AIDS, Yale University, New Haven, CT 06520, USA; 4 Arthur Ashe Institute for Urban Health, Brooklyn, NY 11203, USA; mfraser@arthurasheinstitute.org; 5 Department of Medicine, Cell Biology and Pharmacology, State University of New York Downstate Health Sciences University, Brooklyn, NY 11203, USA; mohamed.boutjdir@downstate.edu; 6 Department of Medicine, New York University Grossman School of Medicine, New York, NY 10016, USA; 7 Cardiovascular Research Program, VA New York Harbor Healthcare System, Brooklyn, NY 11209, USA

**Keywords:** HIV, cardiovascular disease, sexual and gender minorities, sleep, hypertension, mental health, financial toxicity, intersectionality

## Abstract

Ethnic and racial sexual minority men with HIV have a disproportionately higher risk of HIV-related cardiovascular disease (CVD). There is a lack of tailored and culturally salient behavioral interventions to address HIV-related chronic illness in ethnic and racial sexual minority men, and literature on their understanding and awareness of modifiable behavioral risks is limited. The purpose of this study was to assess illness perceptions about HIV and HTN, and describe physical activity, tobacco, and e-cigarette use in Black and Latinx sexual minority men living with HIV. We used the validated Illness Perception Questionnaire-Revised (IPQ-R) to assess perceptions about two interrelated chronic diseases, HIV and CVD. To assess CVD behavioral risk, we assessed physical activity using the International Physical Activity Questionnaire. Tobacco and e-cigarette use were assessed using items from the Behavioral Risk Factor Surveillance System. Sleep difficulties were the most prevalent symptom attributed to HIV, and were statistically associated with fatigue, upset stomach, and loss of strength. Anxiety was reported to be caused by HIV (57%) and HTN (39%). Half of the participants engaged in vigorous activity for 128 min (SD = 135) daily, and 63% engaged in moderate activity for 94 min (SD = 88) daily. Over a third reported current tobacco use and 20% reported current e-cigarette use. This study provides formative data to better understand how Black and Latinx sexual minority men with HIV perceive intersecting chronic illnesses and their engagement in modifiable CVD risk behaviors. Sleep, mental health disparities, and financial hardships were commonly reported. More research is needed to address intersecting chronic illnesses and mental health conditions that are influenced by social positioning over the life course, and impact CVD risk factors. This study was not registered.

## 1. Introduction

Every 34 seconds, an individual dies of cardiovascular disease (CVD) [1]. It is a leading cause of death in the United States (US), and has been for over 100 years [1]. CVD has emerged as a prominent contributor to mortality in people with HIV [2,3]. This is due to chronic inflammation as a result of HIV, which increases CVD risk even when HIV viral load is well controlled [4,5,6,7]. Other literature suggests that combination antiretroviral therapy increases oxidative stress resulting in endothelial dysfunction (impaired vasodilation), unfavorable lipid and metabolic effects, and other cardiovascular symptoms [4,8]. As a result, prominent HIV comorbidities include high blood pressure and type 2 diabetes mellitus [9]. Compared to the general population, CVD risk is 1.5 to 2 times higher in people with HIV, and this risk increases with age [3,10,11]. By the year 2030, an estimated 73% of persons with HIV will be 50 years old or older, about 84% will have at least one chronic condition, and 27% will have three or more conditions [12]. If clinical, public health, and research initiatives towards cardiovascular health equity are not sufficiently addressed by the year 2030, an estimated 78% of persons with HIV will be diagnosed with CVD [12].

The risk of HIV, and CVD as a comorbidity, disproportionately impacts ethnic and racial sexual minority men. Specifically, Black and Latinx gay and bisexual men experience higher rates of HIV and associated comorbid conditions, such as high blood pressure and diabetes [13]. CVD risk is intersectional and is exacerbated by social determinants, external forces, structures, and policies. When these multi-level stressors coalesce, they create barriers to education, safe neighborhoods, resources, and quality health care for those economically, racially, and ethnically marginalized. In sexual and gender minoritized individuals, this is intensified and manifests in repeated experiences of racism, discrimination, transphobia, homophobia, and violence. Over the life course, psychosocial stressors can lead to elevated cardiovascular biomarkers and subsequent higher rates of CVD morbidity and mortality in ethnic and racial sexual and gender-minoritized individuals, continuing to hinder the achievement of cardiovascular health equity [7,14,15,16,17].

Current literature underscores the application of strategic approaches to lifestyle modifications to reduce CVD risk and the impact of inequitable social determinants towards cardiovascular health [18]. Advocating for a shift in perspective from solely treating diseases to incorporating health promotion, the American Heart Association (AHA) updated its cardiovascular health metrics and integrated sleep health as a significant element [19]. Mounting literature on cardiovascular health in sexual and gender minoritized individuals has highlighted significant disparities in heart health and increased CVD risk when compared to heterosexual and cisgender individuals [7,14,17]. Specifically, there is a critical need for research to examine clinical and social determinants, test interventions on cardiovascular risk reduction, integrate technological approaches, and facilitate partnerships with ongoing community engagement in the design and conduct of research, while demonstrating trustworthiness [2,14,17,20,21]. The purpose of this study was to assess illness perceptions about HIV and hypertension (HTN), and describe physical activity, tobacco, and e-cigarette use in Black and Latinx sexual minority men living with HIV.

### Framework

**Figure 1:**
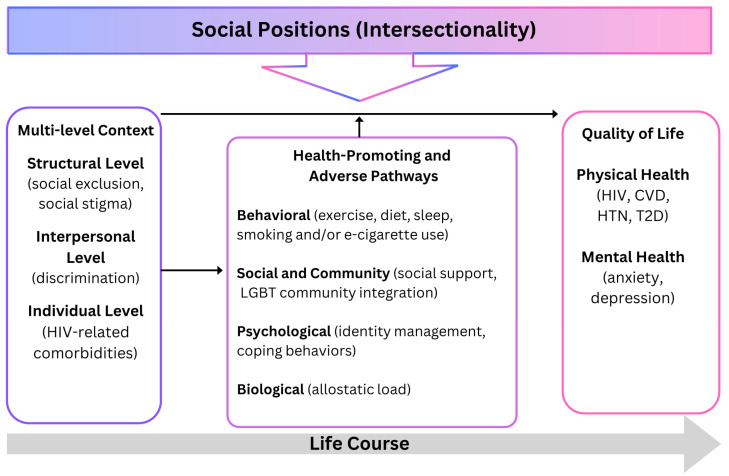
Adapted Health Equity Promotion Model.

This study was guided by an adapted Health Equity Promotion Model (Figure 1) [22]. This is an LGBT-centered model focused on sexual and gender minoritized individuals achieving their full physical and mental health potential. The model describes how intersecting social positions (sexual orientation, gender identity, race, and ethnicity) at the individual and structural levels (discrimination and social exclusion) impact quality of life through physical and mental health along the life course. Multi-level (structural, interpersonal, and individual) factors in the forms of structural forces, systems, and policies continue to marginalize and oppress sexual and gender minoritized individuals and interrupt behavioral pathways (exercise, diet, sleep, and tobacco/e-cigarette use) toward health promotion activities that mitigate health risks (preventative care, diet, physical activity, and smoking cessation). Additionally, lack of social and community cohesion and other psychological factors can influence CVD risk. Intersecting effects of these multilevel stressors, which are driven by social determinants can elevate risk. This is in addition to the cardiometabolic changes that result from HIV [6,13,14]. This model informed our understanding of the perceptions about living with chronic conditions and intersecting health promoting or adverse pathways that influence behavioral CVD risk in Black and Latinx sexual minority men with HIV.

## 2. Materials and Methods

### 2.1. Study Design

Participants were recruited, and cross-sectional descriptive data were collected between May 2021 and October 2022 as part of a formative, community-driven study to map a protocol for a behavioral, CVD prevention intervention in Black and Latinx sexual minority men with HIV [23]. The study was approved by the Yale University Institutional Review Board (#2000031577). All procedures performed were in accordance with the ethical standards of the institutional and national research committee, the 1964 Helsinki Declaration, and its later amendments, or comparable ethical standards. All participants provided informed consent prior to enrollment and engagement in the study. The study methods and results were reported following the Strengthening the Reporting of Observational Studies in Epidemiology (STROBE) Statement for cross-sectional studies [24].

### 2.2. Recruitment

Thirty participants were recruited in partnership with two community-serving organizations in New York City. Both organizations focused on providing supportive services to immigrant, ethnic, racial, low-income, and other minoritized populations. Digital flyers, word of mouth, and snowball sampling were used as recruitment strategies. Eligibility included (1) identifying as non-heterosexual, (2) assigned male sex at birth, (3) ages 30 to 65, (4) ethnic or racial minoritized background, (5) self-reported HIV serostatus positive, and (6) access to the internet. Chronic illness is now diagnosed at earlier stages of life (e.g., ages 18–30) with the highest prevalence at ages 50 and above [25]. Our selected age range was appropriate given the changing age demographic of chronic illness and will provide a thorough description across generations. Using Zoom Video Communications, Inc., eligible participants were screened, consented, and enrolled. Participants completed their survey interview using Zoom in a place they deemed private and acceptable. Interviews were conducted from 60 to 90 min. A translator from the community-based organization was present for participants who were more comfortable answering questions in another language. The translator was a trusted individual who had an established relationship with the participants. A USD $45 Visa gift card was provided as compensation and gratitude for their time.

### 2.3. Measures

#### 2.3.1. Illness Perception Questionnaire-Revised

The revised Illness Perception Questionnaire (IPQ-R) is a validated measure that has been widely used to measure multiple components of illness (i.e., commonly experienced symptoms), cognitive representations of illness (i.e., emotional representations, personal control, treatment control, illness coherence, chronic timeline, cyclical timeline, and consequences), and causal dimensions [26]. The original IPQ-R was adapted for HIV (IPQ-R-HIV) and HTN (IPQ-R-Hypertension) [26]. All participants completed the IPQ-R-HIV, and those with comorbid HTN, forming a subsample, also filled out the IPQ-R-Hypertension.

The IPQ-R-HIV consists of over 80 items that assess perceptions about living with HIV, HIV-related symptoms, and symptoms associated with HIV combination therapy. There are 37 statements about perceptions of living with HIV that are ranked using a 5-point Likert scale (“strongly disagree” to “strongly agree”). Example questions include the following: “The fact that I have HIV causes difficulties for those who are close to me”, and “Having HIV makes me feel anxious”. Other sets of questions assess the severity of symptoms at present by asking, “Are you currently experiencing this symptom as a result of having HIV?” or “taking combination therapy?” A listing of 23 types of symptoms associated with HIV and the use of combination therapy is rated on a 5-point Likert scale (1 = very mild to 5 = very severe). If the response to a symptom is “no” the next symptom is assessed. A higher score indicates the increased severity of a symptom. Examples of a few symptoms listed include pain, sore throat, nausea, and sleep difficulties. The IPQ-R-HIV has high internal consistency (Cronbach’s α = 0.90) [26], and has been tested in samples of sexual minoritized individuals.

The IPQ-R-Hypertension consists of 63 items that assess views about HTN, symptoms, and possible causes of blood pressure symptoms. A total of 23 statements concerning views about HTN are ranked using a 5-point Likert scale (“strongly disagree” to “strongly agree”). For example, statements about individual views on HTN include, “I have the power to influence my high blood pressure”, and “I expect to have this high blood pressure for the rest of my life”. Nineteen common HTN-related symptoms (e.g., pain, breathlessness, fatigue, and headaches) are listed with two follow-up questions. If “yes” is reported for a symptom, the follow-up questions will assess if the symptom is due to having high blood pressure, or the medication for high blood pressure. If the respondent is unsure, they can report that they “don’t know”. Three items assess views about high blood pressure symptoms on a Likert scale. Eighteen items are listed as possible causes of HTN on a 5-point Likert scale. Examples of statements about possible causes of HTN include “hereditary-it runs in my family”, “a germ or virus”, “poor medical care in the past”, and “my own behavior”. The IPQ-R-Hypertension demonstrates good internal consistency (Cronbach’s α = 0.75), good test-retest reliability, and predictive, concurrent, and discriminant validity [26].

#### 2.3.2. International Physical Activity Questionnaire

The International Physical Activity Questionnaire (IPAQ; Short Form) consists of 7-items to assess the number of days per week and the amount of time (in hours and minutes per day) spent on physical activity of different types and intensities (vigorous and moderate) that occurs as part of everyday life over the last seven days. Examples of vigorous physical activity included the following: heavy lifting, aerobics, and fast bicycling. Moderate physical activity examples included carrying light loads and bicycling at a regular pace but did not include walking. Walking included activities for travel, recreation, sport, exercise, or leisure at work or home. The last question assessed time spent sitting on a weekday. The IPAQ (Short Form) possesses psychometrics with good test-retest reliability (Spearman’s ρ around 0.8) and acceptable criterion validity [27].

#### 2.3.3. Behavioral Risk Factor Surveillance System

The Behavioral Risk Factor Surveillance System (BRFSS) was developed by the Centers for Disease Control and Prevention as a tool for assessing self-reported health information on chronic conditions and health risk behaviors, such as obesity, smoking, and alcohol use [28,29]. We focused on items assessing modifiable CVD risk factors, such as tobacco and e-cigarette use. Example questions for tobacco use were, “Have you smoked at least 100 cigarettes in your entire life?” and “During the past 12 months, have you stopped smoking for one day or longer because you were trying to quit smoking?” Two items assessed e-cigarette or other electronic vaping. An example question included, “Have you ever used an e-cigarette or other electronic vaping product, even just one time, in your entire life?”

### 2.4. Data Analysis

Data management and statistical analyses were performed using RStudio version 4.2.1. We conducted descriptive analyses to assess the demographic characteristics of the participants and the frequency of self-reported responses on illness perceptions in binary or Likert scales. IPQ-R-HIV items assessing symptoms contained an initial binary (yes/no) response option. If a symptom was reported as “yes”, the symptom intensity was specified as very mild to very severe. No symptoms, reported as “no” were coded as 0. We examined the association between the most frequently reported symptom (which was eventually identified as sleep difficulties), and other symptoms using Chi-square. Odds ratios (ORs) with 95% confidence intervals (CIs) were estimated and analyses were performed using a two-sided test at a 5% significance level. We calculated the means and standard deviations of time (i.e., days per week, minutes per day) spent on physical activities using the IPAQ. We investigated the proportions of participants who engaged in each type of physical activity of different intensities and whose time spent exercising met the national guidelines for cardiovascular health. We assessed history and current tobacco and e-cigarette use with the proportions of binary responses.

## 3. Results

### 3.1. Participant Characteristics

The majority of participants (70%, n = 21) self-identified ethnically as Latinx. Haitian participants did not self-identify as Latinx. Latino ethnicity refers to persons who have heritage from anywhere in Latin America and the Caribbean, irrespective of race. Although Haiti is part of Latin America, Haitian participants in our study self-identified as Black, but not also Latinx. This may be due to Haiti’s unique history, culture, and primary language of Haitian Creole. The gold standard for reporting race and ethnicity is self-identification [30]. We documented race and ethnicity verbatim as participants identified themselves. HIV duration ranged from 1 to 41 years (Mean = 17.2, SD = 11.1). Participants reported being out of the closet for 25.7 (SD = 14.4) years on average. The preferred gender pronouns were “he/him” for 97% (n = 29) and “she/her” for 3% (n = 1). All participants (N = 30) reported having health insurance and access to care. A total of 97% (n = 29) had a regular provider and were on antiretroviral therapy (Table 1).

### 3.2. Views on Living with Chronic Conditions

We assessed views on living with HIV among all participants (N = 30). Those with comorbid HTN also completed the IPQ-R-Hypertension (43%, n = 13). Two participants, in the sample, reported having diabetes in addition to HIV and HTN, and those who reported comorbid diabetes completed the IPQ-R-Diabetes (7%, n = 2). However, we have not included their results in this study due to the limitations posed by the small sample size. Results are reported using the seven IPQ-R concepts: (1) chronic timeline, (2) cyclical timeline, (3) consequences, (4) personal control, (5) treatment control, (6) illness coherence, and (7) emotional representations [26].

### 3.3. Chronic and Cyclical Timeline

Chronic and cyclical timelines refer to whether an individual perceives their illness time trajectories as chronic or cyclical. Seventy-three percent of participants with HIV (n = 22) and 92% with comorbid HTN (n = 12) believed their condition would improve in time. Greater than 60% of participants with HIV (n = 20) and comorbid HTN (n = 8) reported their condition is likely to be permanent rather than temporary. Fifty-seven percent of participants with HIV (n = 17) and 46% with comorbid HTN (n = 6) reported their illness-related symptoms come and go in cycles.

### 3.4. Consequences

Consequences refer to the influences of their conditions on their lives. Forty-seven percent of participants with HIV (n = 14) and 39% comorbid HTN (n = 5) reported their illness has major consequences on their lives. Eighty percent of participants with HIV (n = 24) and 92% with comorbid HTN (n = 12) reported their illness is a serious condition. Slightly under a third of participants with HIV (n = 9) reported that having HIV strongly affects the way others see them, in contrast to those with comorbid HTN who did not report this perception. Thirty-seven percent of participants reported significant financial consequences due to HIV, whereas no such issues were reported in relation to HTN.

### 3.5. Personal Control

Personal control is related to the individual’s perceived control over their illness. All participants with HIV (N = 30) and 85% with comorbid HTN (n = 11) reported there was a lot they could do to control their condition/symptoms.

### 3.6. Treatment Control

Treatment control refers to how well a treatment controls an illness. Ninety-three percent of participants with HIV (n = 28) and 85% with comorbid HTN (n = 11) reported their treatment can control their illness. Ninety percent of participants with HIV (n = 27) and 69% with comorbid HTN (n = 9) reported the negative effects of their illness could be prevented by their treatment.

### 3.7. Illness Coherence

Illness coherence refers to whether an individual understands their illness. Ninety-three percent of participants with HIV (n = 28) and 77% with comorbid HTN (n = 10) reported having a clear picture or understanding of their condition. Sixty-three percent of all participants with HIV (n = 19) reported the symptoms of their condition are puzzling.

### 3.8. Emotional Representations

Emotional representations refer to the impact of the illness on an individual’s emotional well-being and mental health. Fifty-seven percent of all participants (n = 17) reported HIV makes them feel anxious. Thirty-eight percent with comorbid HTN (n = 5) reported feeling anxious and afraid. About 27% of all participants (n = 8) reported feeling depressed when thinking about having HIV, while one person with comorbid HTN (8%) reported getting depressed due to high blood pressure. Twenty percent (n = 6) reported feeling angry and 30% (n = 9) reported feeling upset due to having HIV, while one (8%) and two (15%) individuals with comorbid HTN felt angry and upset, respectively, because of high blood pressure.

### 3.9. Symptoms Related to HIV and Hypertension

The most prevalent symptoms related to HIV were sleep difficulties (43%, n = 13), stiff joints (37%, n = 11), and fatigue (33%, n = 10). The most frequent symptoms related to combination therapy included upset stomach (20%, n = 6), sleep difficulties (17%, n = 5), stiff joints (17%, n = 5), and altered sensation in hands or feet (17%, n = 5).

The most frequently reported symptoms related to HTN included fatigue (31%, n = 4), breathlessness (23%, n = 3), dizziness (23%, n = 3), or fast heart rate (23%, n = 3). A total of 33 symptom cases were reported from 13 participants with HTN. In more than half of the symptom cases, participants were certain about whether their symptoms were caused by their HTN or antihypertensive medication (52%, n = 17). In two symptom cases (6%), participants reported they were certain that a specific symptom was due to medications but were unsure whether it was related to their HTN. Conversely, in seven symptom cases (21%), the participants were sure that a symptom was associated with HTN but were unsure if it was due to medication. The participants were unsure whether taking medications created their symptoms in 42% of symptom cases (n = 14).

### 3.10. Sleep Difficulties as a Primary Symptom

Many of the symptoms co-occurred with sleep difficulties, which were the most frequently reported symptoms related to HIV. Forty-three percent of all participants (n = 13) had sleep symptoms, and 33% (n = 10) reported moderate to very severe sleep difficulties. Table 2 shows the association between sleep difficulties and other HIV-related symptoms with odds ratios (ORs) and 95% confidence intervals (CIs). Greater proportions of participants experienced sleep difficulties when they were suffering from fatigue (90% vs. 20%, *p* = 0.0003), loss of strength (87.5% vs. 27.3%, *p* = 0.0094), and upset stomach (77.8% vs. 28.6%, *p* = 0.0127) with ORs of 8.75 to 36. More than 60% of participants reporting pain, weight loss, and diarrhea also experienced sleep difficulties. Although CIs are wide due to the small sample size, the ORs are 2.92 to 4.0 for these three symptoms. Only one participant with HTN reported experiencing sleep difficulties, which were related to HTN but unrelated to taking anti-hypertensive medications. This participant also reported HTN-related headaches, impotence, and fast heart rate.

### 3.11. Physical Activity, Tobacco, and E-Cigarette Use

Given the significant impact on CVD risk-related behaviors, such as decreased physical activity, e-cigarette, and nicotine use, we assessed these behaviors along with illness perceptions to provide a comprehensive description of risk in this diverse sample. All participants (N = 30) reported the number of days per week and minutes per day of their physical activity (Table 3). Half of the participants (n = 15) engaged in vigorous physical activity for 512 min per week on average (SD = 703.0), while 19 participants (63%) engaged in moderate physical activity for 333 min per week on average (SD = 398.3). Among the 15 participants who reported vigorous physical activity, 7% (n = 2) engaged in less than 75 min of vigorous physical activity per week, 7% (n = 2) engaged in 75 min or more but less than 150 min per week, and 37% (n = 11) engaged in 150 min or more of vigorous activity per week based on guidelines for physical activity [31]. Among the 19 participants who reported moderate activity, 30% (n = 9) engaged in less than 150 min of moderate physical activity per week, 17% (n = 5) engaged in 150 min or more but less than 300 min per week, and another 17% (n = 5) engaged in 300 min or more of moderate-intensity activity per week. The participants spent 138 (SD = 179) minutes and 385 (SD = 235) minutes daily walking and sitting, respectively. Forty-three percent (n = 13) spent less than 60 min walking daily, while 30% (n = 9) spent more than 60 min but less than 120 min, and 27% (n = 8) spent more than 120 min per day.

Fifty-three percent (n = 16) of participants reported having smoked cigarettes. A third (n = 10) reported current cigarette use and having used e-cigarettes. Twenty percent (n = 6) reported current e-cigarette use. Among current smokers, 50% (n = 5) reported that they had stopped smoking for one day or longer with the purpose of quitting smoking during the past 12 months. A total of 47% of participants (n = 14) never smoked, while 40% (n = 12) reported smoking at least 100 cigarettes in their lives. None of the participants reported current use of chewing tobacco, snuff, or snus.

## 4. Discussion

The purpose of this study was to assess illness perceptions about HIV and HTN, and describe physical activity, tobacco, and e-cigarette use in Black and Latinx sexual minority men living with HIV. This study adds to the literature by capturing the perceptions and CVD behavioral risks of Black and Latinx sexual minority men with HIV, a population that has not had significant achievements in health equity. Our formative data provides early evidence and a foundation for the development of behavioral interventions that are informed and culturally salient.

### 4.1. Perceptions about Chronic Illness

Overall, participants reported complexity in their illness-related symptoms, sleep difficulties, mental health challenges, and subsequent financial adversity. Findings suggested that while the majority of participants view both HIV and HTN as permanent (HIV 67%, HTN 62%) and serious conditions (HIV 80%, HTN 92%), living with HIV may be linked to more significant challenges in disease management and psychosocial difficulties than with HTN. This is evidenced by 57% of participants perceiving their HIV as unpredictable and their symptoms as cyclic, in contrast to 46% for HTN. Moreover, a greater percentage of participants indicated that living with HIV had serious impacts on their lives (47%), compared to those living with HTN (39%). Notably, 30% reported negative effects on interpersonal relationships due to HIV, an issue not reported in relation to HTN. These observations align with the work of Ross and colleagues [32], who found that patients with HTN, but not HIV, reported lower scores of perceived significant consequences resulting from high blood pressure. This suggests that the chronic nature of HIV, coupled with the stigma associated with the virus and the severity of the condition when not virally suppressed, may lead to greater adverse consequences than those from more common and less stigmatized chronic conditions like HTN.

Participants hold positive views of their conditions, yet they concurrently encounter challenges in managing their illnesses. Participants were optimistic that their illnesses would improve over time (HIV 73%, HTN 92%), and they considered their conditions (HIV and/or HTN) to be controlled with treatment (HIV 93%, HTN 85%). Participants reported having enough understanding about their conditions and perceived the availability of treatment and its effectiveness in prevention. However, a relatively lower percentage of respondents perceived the long-term effectiveness of the treatment in curing their illness (HIV 47%, HTN 54%). Sixty-three percent of those with HIV still considered their condition puzzling, which suggests varying levels of HIV knowledge and behavioral self-management. It also suggests that living with HIV-associated symptoms, comorbidities, or both can be a daily challenge.

### 4.2. Financial Toxicity

An unanticipated finding from our data was that 37% of participants reported on the IPQ-R serious financial consequences related to having HIV, but not due to HTN. Financial toxicity is a serious consideration when having one or more chronic conditions. One study reported that Black individuals with clinical or behavioral CVD risk factors reported higher lifetime healthcare expenditures [33]. Nearly 50% of this study’s participants had an annual household income of below USD 20,000, and for some, their income was as low as USD 6000. National poverty guidelines set the annual income at USD 14,580 in 48 states and the District of Columbia [34]. The annual gross income in New York State is USD 26,973 for a household of one person [35], which suggests that persons with HIV are more likely at risk of experiencing financial hardships than persons without HIV.

### 4.3. Sleep and Mental Health

Sexual and gender minoritized individuals have a higher lifetime prevalence of suicidality, depression, anxiety, and consequent tobacco and substance use behaviors compared to heterosexual and cisgender persons [13]. In this study, participants reported internalizing symptoms such as anxiety and depression related to HIV (57% and 27%, respectively) and HTN-related anxiety (39%). Having HIV heightens the risk of emotional dysregulation, creating feelings of anger (20%) and upset (30%). The literature suggests that mental health is associated with sleep quality [36]. Sleep deficiency can lead to cardiovascular and metabolic diseases such as HTN, diabetes, and obesity [37,38]. According to the data from the National Health Interview Survey, 15–18% of adults experienced sleep disturbances such as difficulties in falling asleep and maintaining quality sleep [39]. Findings from our study included a much greater percentage (43%) reporting sleep difficulties, even allowing for a more general term used in the measure to assess sleep-related symptoms, highlighting the connection between sleep and mental health disparities in our sample of Black and Latinx sexual minority men with HIV. This is an important consideration as disparities in sleep and mental health aggravate CVD risk burdens. It also highlights the interrelatedness between sleep health, mental health, and heart health [40].

Nonetheless, addressing mental health equity in sexual minoritized populations is a pressing issue with significant structural- and individual-level challenges. In 2016, approximately 12.6% of mental health facilities in the US offered LGBT-specific programs [41]. As of 2020, approximately 27% of mental health facilities offer treatment programs for LGBT clients [42], leaving a considerable gap in access and availability despite the well-documented need. Mental health stigma and its interrelation with HIV, sexual orientation, gender identity, race, and ethnicity further complicate disparities. New research modalities and approaches are needed to enhance mental health equity in sexual and gender minoritized populations. One study used minority stress-focused LGBQ-affirmative cognitive behavioral therapy to address mental and behavioral health concerns in sexual minority men [43,44]. Findings suggested a statistically significant moderator of the CBT intervention efficacy was Black or Latinx race and ethnicity [43,44]. In that study, Black and Latinx individuals experienced greater reductions in anxiety and substance use, compared to non-Latinx, White participants [43,44]. Another study delivered problem-solving therapy (PST) to individuals using an artificial intelligence, virtual voice-based coach [45,46]. Findings showed that virtual, voice-based PST reduced symptoms of depression and anxiety, especially in ethnic, racial, and socioeconomically marginalized individuals with a college education or less [45,46]. Further research using remote and other innovative modalities is needed to address mental health outcomes in sexual and gender minoritized individuals.

### 4.4. Physical and Sedentary Activity and Nicotine Use

The effects of inequitable structures, forces, systems, and policies on the health and well-being of sexual minoritized individuals can result in maladaptive coping, such as nicotine use, that may increase health risk [47]. The average time spent engaging in sedentary activities by people in the US is approximately 7.7 h daily [48]. The participants in this study spent approximately 6.4 h (385 min, SD = 3.5) sitting daily. Twenty-seven percent (n = 8) of participants reported spending eight hours or longer per day sitting. Greater than 50% of participants did not meet the minimum recommendations for moderate and vigorous physical activity [31,48]. Guidelines for physical activity recommend that adults get between 150 to 300 min per week of moderate-intensity aerobic, or cardio activity, or 75 to 150 min per week of vigorous activity [31,48,49]. The proportion of individuals reporting current cigarette use in the US was 9.7% for heterosexuals, and 16.0% for lesbian, gay, and bisexual persons [1]. In this study, nicotine use among the participants was 33%. Among the participants who ever used e-cigarettes, over half of participants (60%, n = 6) continued to use e-cigarettes following their initial exposure. These findings align with previous research that sexual minoritized individuals are at a higher risk of substance use and are concordant with the work of colleagues examining CVD risk behaviors in sexual and gender minoritized populations [13]. Nonetheless, half of the participants using nicotine in this study attempted cessation. All of the participants were engaged in daily light-intensity physical activity, such as walking, with approximately 80% walking for at least four days per week, up to every day. We believe this is due to living in New York City, where walking is a common practice.

## 5. Limitations

While the study provides new insights into illness perceptions and CVD risk in Black and Latinx sexual minority men with HIV, the following limitations should be considered. First, this study used validated measures that were similar in format but did not consist of equivalent items for each dimension. These discrepancies limited the ability to compare the results of illness perceptions according to chronic condition type. Second, the data were gathered cross-sectionally through self-reported surveys. For individuals living with HIV and comorbidities, distinguishing between symptoms unique to HIV and those resulting from comorbid conditions may be difficult due to their overlapping nature. Consequently, parsing out symptomatology and consequences attributed solely to either HIV or comorbidities may not be discernible. Third, responses to five or six emotional representation items in the IPQ-R-Hypertension and IPQ-R-HIV were interpreted as proxy indicators of mental health issues. However, these subitems, while contributing to the broader illness perception assessment, lack diagnostic and confirmatory validity for conditions such as depression and anxiety. Therefore, this study’s findings pertaining to mental health should be interpreted with caution and may not be definitive. Lastly, this study included 30 participants, 13 of whom were comorbid with HTN and two of whom were comorbid with diabetes. We sought to explore the illness perceptions associated with comorbid diabetes as well as HIV and HTN; however, due to the interpretive limitations imposed by the small sample size of participants with diabetes (n = 2), these results are not reported in this study. As the small sample size and a non-probabilistic sampling technique limit generalizability and also lack the power for inferential statistics, further research involving larger sample sizes and random sampling is essential to gain a deeper understanding of the experiences associated with HIV and its comorbidities, as well as to investigate the underlying associations among illness perceptions, symptoms, and health behaviors in sexual minority men of color with HIV.

## 6. Implications for Research and Clinical Nursing Practice

In 2019, the US National Institutes of Health convened a workshop to examine the comorbidities, coinfections, and complications associated with HIV [50]. The discussions suggested that the underlying causes and mechanisms of HIV-related conditions leading to multimorbidity may differ significantly from those observed in HIV-negative individuals across the lifespan [50,51]. Additionally, the chronic immune activation and dysfunction seen in managed HIV are driven by shared immune pathways, as well as microbiome/virome contributions to inflammation and accentuated aging [50,52]. Consequently, further research is warranted to investigate biomarkers, mechanisms, and interventions for a better understanding of HIV-related comorbidities across the lifespan [50]. Nevertheless, the exploratory, descriptive analysis of this study provides unique insights into the health and illness-related perceptions and behaviors of Black and Latinx sexual minority men living with HIV, which is a crucial first step towards mitigating the onset and impact of these conditions.

Increased focus on preventive screenings in clinical and community settings is essential, as HIV-related comorbidities such as cardiovascular disease, hypertension, and type 2 diabetes are prevalent. These conditions share common behavioral risk factors, including poor nutrition, tobacco or e-cigarette use, and a sedentary lifestyle. An effective approach is to ensure that patients are equipped with the necessary knowledge and resources to make informed health decisions, collaborating closely with their healthcare providers. Nursing is the most trusted profession [53], and nurses have the responsibility to foster meaningful and lasting improvements in the well-being of their patients.

## 7. Conclusions

This study provided descriptive data to better understand how Black and Latinx sexual minority men with HIV perceive intersecting chronic illnesses and their engagement in modifiable CVD risk behaviors, as this has not been fully understood in the extant literature. Sleep, mental health disparities, and financial hardships were commonly reported. More research is needed to address intersecting chronic illness and mental health conditions that are influenced by social positioning over the life course, and impact CVD risk factors.

## Figures and Tables

**Table 1:** Participant Characteristics.

Variables (N = 30)	Mean (SD)	Range
Age (year)	47.47 (12.53)	30–65
		N (%)
Race	Black	3 (10)
	Haitian ^a^	4 (13.3)
	Mixed or biracial	13 (43.3)
	Unspecified ^b^	1 (3.3)
Latinx ^c^		21 (70)
US Born		13 (43.3)
Highest level of academic attainment		
	High school or GED ^d^	14 (46.7)
	Some college	4 (13.3)
	2- or 4-year degree	6 (20)
	Graduate degree	3 (10)
	Other	3 (10)
Employment	Part-time	5 (16.7)
	Full-time	7 (23.3)
	Unemployed	14 (46.7)
	Retired	3 (10)
	Disabled	1 (3.3)
Annual income		
	Less than $20,000	14 (46.7)
	$20,001–$40,000	10 (33.3)
	$40,001–$60,000	6 (20)
	$60,001 and above	0 (0)
Relationship status	Partnered	9 (30)
	Single	21 (70)

^a^ Self-identification is the gold standard for documenting race and ethnicity. As Haitian participants did not self-report their identity as Latinx, they were not included in the Latinx ethnicity and reported separately. ^b^ This person did not want to specify a race as they just considered themselves Latinx. ^c^ Latinx ethnicity included those who racially identified as either Black or White. ^d^ GED general educational development, equivalent to a high school diploma.

**Table 2:** Associations between Sleep Difficulties and Symptoms related to HIV (N = 30).

		Sleep Difficulties		% of Sleep Difficulties	Odds Ratio [95% CI] *	*p*-Value
		Yes	No			
**Stiff Joints**	Yes	6	5	54.5%	2.06 [0.45, 9.30]	0.3457
	No	7	12	36.8%		
**Fatigue**	Yes	9	1	90.0%	36.00 [3.47, 373.19]	0.0003
	No	4	16	20.0%		
**Upset Stomach**	Yes	7	2	77.8%	8.75 [1.40, 54.80]	0.0127
	No	6	15	28.6%		
**Diarrhea**	Yes	6	3	66.7%	4.00 [0.76, 20.96]	0.0913
	No	7	14	33.3%		
**Altered Sensation in Hands or Feet**	Yes	5	4	55.6%	2.03 [0.42, 9.89]	0.3765
	No	8	13	38.1%		
**Pain**	Yes	5	3	62.5%	2.92 [0.55, 15.56]	0.2014
	No	8	14	36.4%		
**Weight Loss**	Yes	5	3	62.5%	2.92 [0.55, 15.56]	0.2014
	No	8	14	36.4%		
**Loss of Strength**	Yes	7	1	87.5%	18.67 [1.88, 185.41]	0.0094
	No	6	16	27.3%		

* *CI* = confidence interval.

**Table 3:** Physical Activity Engagement.

(N = 30)	Vigorous	Moderate	Walking	Sitting
Engagement	N (%)	N (%)	N (%)	N (%)
**Days/week**				
**0**	15 (50)	11 (36.7)	0 (0)	
**1**	1 (3.3)	3 (10)	1 (3.3)	N/A
**2**	5 (16.7)	5 (16.7)	2 (6.7)	
**3**	1 (3.3)	4 (13.3)	3 (10)	
**4**	1 (3.3)	1 (3.3)	2 (6.7)	
**5**	6 (20)	2 (6.7)	6 (20)	
**6**	0 (0)	0 (0)	2 (6.7)	
**7**	1 (3.3)	4 (13.3)	14 (46.7)	
	**Mean (SD)** **N = 15**	**Mean (SD)** **N = 19**	**Mean (SD)** **N = 30**	**Mean (SD)** **N = 30**
**Days/week**	3.7 (1.8)	3.5 (2.2)	5.4 (1.9)	N/A
**Minutes/day**	128 (134.6)	94.2 (88.1)	137.8 (178.5)	384.8 (234.8)
**Minutes/week**	512 (703)	333.4 (398.3)	883.3 (1274.6)	N/A

## Data Availability

The data presented may be available upon reasonable request to the corresponding author.

## Public Involvement Statement

We partnered with two community-based organizations. CBO administrators were involved with priority setting, planning acceptable methods of consenting, and data collection, assisted with conducting the research by translating in Spanish and Haitian Creole, and facilitated disseminating findings back to the participant community. They also provided guidance and direction for future studies. The PI provided routine study updates, checked in for feedback as the study progressed, and created dissemination posters in English, Haitian Creole, and Spanish, with feedback from the community partners on how the information should be presented and translated.

## Guidelines and Standards Statement

The study methods and results were reported following the Strengthening the Reporting of Observational Studies in Epidemiology (STROBE) Statement for cross-sectional studies.

## References

[1] Martin S.S., Aday A.W., Almarzooq Z.I., Anderson C.A., Arora P., Avery C.L., Baker-Smith C.M., Gibbs B.B., Beaton A.Z., Boehme A.K. (2024). 2024 Heart Disease and Stroke Statistics: A Report of US and Global Data From the American Heart Association. Circulation.

[2] Diaz C.L., Shah N.S., Lloyd-Jones D.M., Khan S.S. (2021). State of the Nation’s Cardiovascular Health and Targeting Health Equity in the United States A Narrative Review. JAMA Cardiol..

[3] Hsue P.Y., Waters D.D. (2018). Time to Recognize HIV Infection as a Major Cardiovascular Risk Factor. Circulation.

[4] Titanji B., Gavegnano C., Hsue P., Schinazi R., Marconi V.C. (2020). Targeting Inflammation to Reduce Atherosclerotic Cardiovascular Risk in People With HIV Infection. J. Am. Heart Assoc..

[5] American Heart Association News As HIV Patients Live Longer, Heart Disease Might Be Their Next Challenge. https://www.heart.org/en/news/2019/06/03/as-hiv-patients-live-longer-heart-disease-might-be-their-next-challenge.

[6] Feinstein M.J., Hsue P.Y., Benjamin L.A., Bloomfield G.S., Currier J.S., Freiberg M.S., Grinspoon S.K., Levin J., Longenecker C.T., Post W.S. (2019). Characteristics, Prevention, and Management of Cardiovascular Disease in People Living With HIV: A Scientific Statement From the American Heart Association. Circulation.

[7] Gosiker B.J., Lesko C.R., Rich A.J., Crane H.M., Kitahata M.M., Reisner S.L., Mayer K.H., Fredericksen R.J., Chander G., Mathews W.C. (2020). Cardiovascular disease risk among transgender women living with HIV in the United States. PLoS ONE.

[8] Dau B., Holodniy M. (2008). The Relationship Between HIV Infection and Cardiovascular Disease. Curr. Cardiol. Rev..

[9] Bygrave H., Golob L., Wilkinson L., Roberts T., Grimsrud A. (2020). Let’s talk chronic disease: Can differentiated service delivery address the syndemics of HIV, hypertension and diabetes?. Curr. Opin. HIV AIDS.

[10] Losina E., Hyle E.P., Borre E.D., Linas B.P., E Sax P., Weinstein M.C., Rusu C., Ciaranello A.L., Walensky R.P., A Freedberg K. (2017). Projecting 10-year, 20-year, and Lifetime Risks of Cardiovascular Disease in Persons Living With Human Immunodeficiency Virus in the United States. Clin. Infect. Dis..

[11] Shah A.S., Stelzle D., Lee K.K., Beck E.J., Alam S., Clifford S., Longenecker C.T., Strachan F., Bagchi S., Whiteley W. (2018). Global Burden of Atherosclerotic Cardiovascular Disease in People Living With HIV: Systematic Review and Meta-Analysis. Circulation.

[12] Smit M., Brinkman K., Geerlings S., Smit C., Thyagarajan K., van Sighem A., de Wolf F., Hallett T.B. (2015). Future challenges for clinical care of an ageing population infected with HIV: A modelling study. Lancet Infect. Dis..

[13] Caceres B.A., Ancheta A.J., Dorsen C., Newlin-Lew K., Edmondson D., Hughes T.L. (2022). A population-based study of the intersection of sexual identity and race/ethnicity on physiological risk factors for CVD among U.S. adults (ages 18–59). Ethn. Health.

[14] Caceres B.A., Streed C.G.S., Corliss H.L., Lloyd-Jones D.M., Matthews P.A., Mukherjee M., Poteat T., Rosendale N., Ross L.M., On behalf of the American Heart Association Council on Cardiovascular (2020). Assessing and Addressing Cardiovascular Health in LGBTQ Adults: A Scientific Statement From the American Heart Association. Circulation.

[15] Rosati F., Williams D.P., Juster R.-P., Thayer J.F., Ottaviani C., Baiocco R. (2021). The Cardiovascular Conundrum in Ethnic and Sexual Minorities: A Potential Biomarker of Constant Coping With Discrimination. Front. Neurosci..

[16] Thayer J.F., Carnevali L., Sgoifo A., Williams D.P. (2020). Angry in America: Psychophysiological Responses to Unfair Treatment. Ann. Behav. Med..

[17] Streed C.G., Beach L.B., Caceres B.A., Dowshen N.L., Moreau K.L., Mukherjee M., Poteat T., Radix A., Reisner S.L., Singh V. (2021). Assessing and Addressing Cardiovascular Health in People Who Are Transgender and Gender Diverse: A Scientific Statement From the American Heart Association. Circulation.

[18] Commodore-Mensah Y., Loustalot F., Himmelfarb C.D., Desvigne-Nickens P., Sachdev V., Bibbins-Domingo K., Clauser S.B., Cohen D.J., Egan B.M., Fendrick A.M. (2022). Proceedings From a National Heart, Lung, and Blood Institute and the Centers for Disease Control and Prevention Workshop to Control Hypertension. Am. J. Hypertens..

[19] Lloyd-Jones D.M., Allen N.B., Anderson C.A., Black T., Brewer L.C., Foraker R.E., Grandner M.A., Lavretsky H., Perak A.M., Sharma G. (2022). Life’s Essential 8: Updating and Enhancing the American Heart Association’s Construct of Cardiovascular Health: A Presidential Advisory From the American Heart Association. Circulation.

[20] Ramos S.R., Warren R., Shedlin M., Melkus G., Kershaw T., Vorderstrasse A. (2019). A Framework for Using eHealth Interventions to Overcome Medical Mistrust Among Sexual Minority Men of Color Living with Chronic Conditions. Behav. Med..

[21] Agarwala A., Patel J., Stephens J., Roberson S., Scott J., Beckie T., Jackson E.A., on behalf of the American Heart Association Prevention Science Committee of the Council on Epidemiology and Prevention and Council on Cardiovascular and Stroke Nursing, Council on Clinical Cardiology, Council on Lifestyle and Cardiometabolic Health (2023). Implementation of Prevention Science to Eliminate Health Care Inequities in Achieving Cardiovascular Health: A Scientific Statement From the American Heart Association. Circulation.

[22] Fredriksen-Goldsen K.I., Simoni J.M., Kim H.-J., Lehavot K., Walters K.L., Yang J., Hoy-Ellis C.P., Muraco A. (2014). The health equity promotion model: Reconceptualization of lesbian, gay, bisexual, and transgender (LGBT) health disparities. Am. J. Orthopsychiatry.

[23] Ramos S.R., Fraser M., Araya F., Kim H.Y., Parrilla J.A.S., Sy K.M., Nagpal R.T., Camacho-Rivera M., Boutjdir M. (2022). Community-Engaged Intervention Mapping for Cardiovascular Disease Prevention in Black and Latinx Sexual Minority Men With HIV in New York City: Protocol for a Web-Based Mixed Methods Study. JMIR Res. Protoc..

[24] Von Elm E., Altman D.G., Egger M., Pocock S.J., Gøtzsche P.C., Vandenbroucke J.P., Initiative S. (2007). The Strengthening the Reporting of Observational Studies in Epidemiology (STROBE) statement: Guidelines for reporting observational studies. Lancet.

[25] Fox S., Duggan M., Rainie L., Purcell K. (2013). The Diagnosis Difference: A Portrait of the 45% of US Adults Living with Chronic Health Conditions.

[26] Moss-Morris R., Weinman J., Petrie K., Horne R., Cameron L., Buick D. (2002). The Revised Illness Perception Questionnaire (IPQ-R). Psychol. Health.

[27] Craig C.L., Marshall A.L., Sjöström M., Bauman A.E., Booth M.L., Ainsworth B.E., Pratt M., Ekelund U.L., Yngve A., Sallis J.F. (2003). International Physical Activity Questionnaire: 12-Country Reliability and Validity. Med. Sci. Sports Exerc..

[28] Pierannunzi C., Hu S.S., Balluz L. (2013). A systematic review of publications assessing reliability and validity of the Behavioral Risk Factor Surveillance System (BRFSS), 2004–2011. BMC Med Res. Methodol..

[29] Yore M.M., Ham S.A., Ainsworth B.E., Kruger J., Reis J.P., Kohl H.W., Macera C.A. (2007). Reliability and Validity of the Instrument Used in BRFSS to Assess Physical Activity. Med. Sci. Sports Exerc..

[30] Office of Management and Budget Provisional Guidance on the Implementation of the 1997 Standards for Federal Data on Race and Ethnicity. https://www.federalregister.gov/documents/2001/01/16/01-1132/provisional-guidance-on-the-implementation-of-the-1997-standards-for-federal-data-on-race-and.

[31] Piercy K.L., Troiano R.P., Ballard R.M., Carlson S.A., Fulton J.E., Galuska D.A., George S.M., Olson R.D. (2018). The Physical Activity Guidelines for Americans. JAMA.

[32] Ross S., Walker A., MacLeod M.J. (2004). Patient compliance in hypertension: Role of illness perceptions and treatment beliefs. J. Hum. Hypertens..

[33] Khera R., Kondamudi N., Liu M., Ayers C., Spatz E.S., Rao S., Essien U.R., Powell-Wiley T.M., Nasir K., Das S.R. (2023). Lifetime healthcare expenses across demographic and cardiovascular risk groups: The application of a novel modeling strategy in a large multiethnic cohort study. Am. J. Prev. Cardiol..

[34] Department of Health and Human Services Annual Update of the HHS Poverty Guidelines. https://www.federalregister.gov/documents/2023/01/19/2023-00885/annual-update-of-the-hhs-poverty-guidelines.

[35] New York State Department of Health 2023–2024 Federal Income Guidelines. https://www.health.ny.gov/prevention/nutrition/wic/income_guidelines.htm.

[36] Scott A.J., Webb T.L., James M.M.-S., Rowse G., Weich S. (2021). Improving sleep quality leads to better mental health: A meta-analysis of randomised controlled trials. Sleep Med. Rev..

[37] Luyster F.S., Strollo P.J., Zee P.C., Walsh J.K. (2012). Sleep: A Health Imperative. Sleep.

[38] Lin C., Chien W., Chung C., Wu F. (2018). Risk of type 2 diabetes in patients with insomnia: A population-based historical cohort study. Diabetes/Metabolism Res. Rev..

[39] Adjaye-Gbewonyo D., Ng A.E., Black L.I. Sleep Difficulties in Adults: United States, 2020. https://stacks.cdc.gov/view/cdc/117490.

[40] Ramos S.R., Gaffey A.S.R., Kang B., McCall T. How Sleep Affects Mind, Body, and Heart Health. https://www.sbm.org/healthy-living/how-sleep-affects-mind-body-and-heart-health.

[41] Williams N.D., Fish J.N. (2020). The availability of LGBT-specific mental health and substance abuse treatment in the United States. Health Serv. Res..

[42] Statista Mental Health Facilities Offering Programs for Specific Client Groups U.S. 2020. Percentage of U.S.. https://www.statista.com/statistics/712859/mental-health-facilities-offering-treatment-programs-for-specific-client-groups-in-us/.

[43] Keefe J.R., Rodriguez-Seijas C., Jackson S.D., Bränström R., Harkness A., Safren S.A., Hatzenbuehler M.L., Pachankis J.E. (2023). Moderators of LGBQ-affirmative cognitive behavioral therapy: ESTEEM is especially effective among Black and Latino sexual minority men. J. Consult. Clin. Psychol..

[44] Pachankis J.E., Harkness A., Maciejewski K.R., Behari K., Clark K.A., McConocha E., Winston R., Adeyinka O., Reynolds J., Bränström R. (2022). LGBQ-affirmative cognitive-behavioral therapy for young gay and bisexual men’s mental and sexual health: A three-arm randomized controlled trial. J. Consult. Clin. Psychol..

[45] Kannampallil T., Ronneberg C.R., E Wittels N., Kumar V., Lv N., Smyth J.M., Gerber B.S., A Kringle E., A Johnson J., Yu P. (2022). Design and Formative Evaluation of a Virtual Voice-Based Coach for Problem-solving Treatment: Observational Study. JMIR Form. Res..

[46] Kannampallil T., Ajilore O.A., Lv N., Smyth J.M., Wittels N.E., Ronneberg C.R., Kumar V., Xiao L., Dosala S., Barve A. (2023). Effects of a virtual voice-based coach delivering problem-solving treatment on emotional distress and brain function: A pilot RCT in depression and anxiety. Transl. Psychiatry.

[47] Merschel M. Heart Health Report Aims to Bolster Research, Boost Care for LGBTQ Patients. https://www.heart.org/en/news/2020/10/08/heart-health-report-aims-to-bolster-research-boost-care-for-lgbtq-patients.

[48] U.S. Department of Health and Human Services Physical Activity Guidelines for Americans, 2nd edition.

[49] American Heart Association American Heart Association Recommendations for Physical Activity in Adults and Kids. https://www.heart.org/en/healthy-living/fitness/fitness-basics/aha-recs-for-physical-activity-in-adults.

[50] Pahwa S., Deeks S., Zou S., Tomitch N.M., Miller-Novak L., Caler E., Justice A., Sacktor N., Gabuzda D., Hunt P.W. (2021). NIH Workshop on HIV-Associated Comorbidities, Coinfections, and Complications: Summary and Recommendation for Future Research. Am. J. Ther..

[51] Gabuzda D., Jamieson B.D., Collman R.G., Lederman M.M., Burdo T.H., Deeks S.G., Dittmer D.P., Fox H.S., Funderburg N.T., Pahwa S.G. (2020). Pathogenesis of Aging and Age-related Comorbidities in People with HIV: Highlights from the HIV ACTION Workshop. Pathog. Immun..

[52] Lagathu C., Cossarizza A., Béréziat V., Nasi M., Capeau J., Pinti M. (2017). Basic science and pathogenesis of ageing with HIV: Potential mechanisms and biomarkers. AIDS.

[53] Walker A. Nursing Ranked as the Most Trusted Profession for 22nd Year in a Row. Nursing.org. https://nurse.org/articles/nursing-ranked-most-honest-profession/.

